# Shared decision-making intervention regarding dialysis modality in patients with CKD stage 5

**DOI:** 10.1097/MD.0000000000033695

**Published:** 2023-05-12

**Authors:** Young-Ki Lee, Yang-Hyeon Kim, Do-Hyoung Kim, Jin-Heog Kim, Jeong-Hwan Lee, Ji Hyeon Park, Gang-Jee Ko, Won-Min Hwang, Hyo-Wook Gil, Young-Sun Kang, Kyu-Bok Jin, Jun-Young Do, Se-Joong Kim, Beom-Seok Kim, Ho Sik Shin

**Affiliations:** a Department of Internal Medicine, Kangnam Sacred Heart Hospital, Hallym University College of Medicine, Seoul, Republic of Korea; b Renal Division, Department of Internal Medicine, Gospel Hospital, Kosin University College of Medicine, Busan, South Korea; c Transplantation Research Institute, Kosin University College of Medicine, Busan, South Korea; d Department of Bigdata and Applied Statistics, College of Science and Technology, Dongguk University, Gyeongju, Republic of Korea; e Department of Internal Medicine, Seoul National University College of Medicine, Seoul, Republic of Korea; f Department of Internal Medicine, National Police Hospital, Seoul, Republic of Korea; g Department of Internal Medicine, Korea University College of Medicine, Seoul, Republic of Korea; h Department of Internal Medicine, Konyang University Hospital, Daejeon, Republic of Korea; i Soonchunhyang University Cheonan Hospital, Cheonan, Korea; j Korea University Ansan Hospital, Seoul, Korea; k Division of Nephrology, Department of Internal Medicine, Keimyung University Dongsan Hospital, Keimyung University Kidney Institute, Keimyung University School of Medicine, Daegu, Republic of Korea; l Yeungnam University Hospital, Daegu, Korea; m Department of Internal Medicine, Seoul National University Bundang Hospital, Seoul, Korea; n Yonsei University College of Medicine, Seoul, Korea.

**Keywords:** patient-centered care, peritoneal dialysis, prognosis, quality of life, shared decision-making

## Abstract

Patients with kidney failure must make complicated decisions about the dialysis modalities used either at home or in-hospital. Different options have varying levels of impact on patients’ physical and psychological conditions and their social life. The purpose of this study was to evaluate the implementation of an intervention designed to achieve shared decision making (SDM) in patients’ options for dialysis.

**Methods:**

SDM was performed after consent was written for stage 5 chronic kidney disease patients before dialysis, and 435 cases were performed in 408 patients from December 16, 2019 to June 30, 2021. Among these, 101 patients were compared by SDM measurement scale, patient satisfaction, disease recognition scale survey, and dialysis method.

**Results:**

The average age of participants was 56 years, with a gender composition of 55 males (54.5%) and 46 females (45.5%). Following SDM, the final dialysis methods decided upon by patients and clinicians were peritoneal dialysis (67 patients, 66.3%), hemodialysis (22 patients, 21.8%), and kidney transplantation (1 patient, 1.0%).

**Conclusions:**

Among participating patients, SDM was effective when used to decide on dialysis treatment, and patients were satisfied with the dialysis method decision process. On the disease awareness scale, those who participated in this project had relatively high positive and low negative perceptions, so it can be concluded that SDM was relatively effective. The implementation of SDM was helpful in selecting patients’ best dialysis methods, and SDM scale results were higher in the peritoneal dialysis group than in the hemodialysis group.

## 1. Introduction

The number of people undergoing maintenance dialysis globally has increased dramatically. Chronic kidney disease (CKD) is not only a public health issue but also creates an economic burden. Patients whose advanced CKD is approaching ESRD face complex medical decision-making regarding the type of medical therapy they wish to pursue. Patients with kidney failure must make complicated decisions about dialysis modalities used either at home or in-hospital. Different options have varying levels of impact on patients’ physical and psychological conditions and their social life. Effective interventions to guide patients in decision-making include decision aids and shared decision-making. There is growing interest in developing, implementing, and further strengthening the quality of decision support provided to patients and families living with CKD.

This study aims to evaluate the implementation of an intervention designed to achieve shared decision making (SDM) in patients’ options for dialysis. The study’s specific objectives are to measure decision quality as indicated by patients’ knowledge, readiness, and achieved preferences and to determine if patients experienced SDM according to design.

## 2. Methods

A mixed methods descriptive study was conducted using both questionnaires and semi-structured interviews. Eligible participants were adults with kidney failure confronting decisions about dialysis modalities. The proposed intervention, based on the three-talk model for SDM, consisted of a patient decision aid and decision coaching meetings provided by trained dialysis coordinators.

A total of 408 patients with CKD ahead of dialysis participated in SDM. Among patients participating in the at-home management pilot project for peritoneal dialysis (PD) patients in 8 hospitals in Korea, a SDM participation survey was conducted in 101 patients who received educational counseling I (IB511) for determination of dialysis type. Concordance between knowledge, decisions, and preferences in patients was calculated to measure decision quality. Interview transcripts were analyzed qualitatively. Each question was given a maximum stain of 4 or more points as an indicator of positive answers. And We presented the percentage of the answers above 4 points.

Since the survey was conducted anonymously, approval from the ethics committee is not required.

## 3. Results

### 3.1. Status of SDM in the PD at-home management pilot project

Status assessment is made according to a review of decision data from December 16, 2019 to December 31, 2021, provided by the Home Medical Fee Division of the Medical Fee Office of the Health Insurance Review and Assessment Service, IB511 for determination of dialysis type in patients with kidney failure.

Home management services were implemented by 408 people out of 557 qualifying candidates, which accounted for 0.9% of the total number of 59,562 requests for home management services. Characteristics of patients who received educational counseling I for dialysis type confirmation are shown in Table 1.

**Table 1:** Basic characteristics of patients with educational counseling for dialysis type confirmation.

		Shared decision making (IB511)
Number		408
Age (yr)		58.9 ± 13.3
Sex	Male	247 (60.5%)
	Female	161 (39.5%)
Training sessions (times)		1.07 ± 0.26
Education target	Patient	275 (67.6%)
	Patient & Guardian	132 (32.4%)
Educational materials provided	Yes	380 (93.1%)
	No	28 (6.9%)
Patient understanding		3.40 ± 0.98

### 3.2. Participating patient survey

A SDM participation survey was conducted among 101 patients in the at-home management pilot project for PD patients at 8 domestic hospitals who received IB511 to determine dialysis type. The average age of participants was 56 years old, with a gender composition of 55 males (54.5%) and 46 females (45.5%). Following SDM, the final dialysis methods decided upon by patients and clinicians were PD (67 patients, 66.3%), hemodialysis (HD, 22 patients, 21.8%), and kidney transplantation (1 patient, 1.0%), with 11 patients (10.9%) remaining undecided.

#### 3.2.1. SDM measurement scale.

(Distribution of scores of 4 points or higher – indicating positive answers of “yes, very much” – on a 5-point scale.)

On 5 out of 9 items to measure the effectiveness of SDM for patients confronting decisions about dialysis treatment, the score distribution was over 90% with positive answers of 4 points or higher on a 5-point scale. Thus SDM is determined to be effective for patients confronting decisions about dialysis treatment in Table 2.

**Table 2:** Shared decision making measurement scale results.

Questionnaire items	4 Points or higher
1) My attending physician made it clear that a decision was needed.	97.3%
2) My attending physician wanted to know exactly how I was involved in making the decision.	87.4%
3) My attending physician said that there were several options to choose from depending on my health condition.	89.2%
4) My doctor explained in detail the pros and cons of each treatment option.	92.8%
5) My attending physician helped me understand the information.	93.7%
6) My doctor asked what my preferred treatment was.	91.0%
7) My doctor and I thoroughly evaluated the different options for treatment.	84.6%
8) My doctor and I chose treatment together.	88.3%
9) My doctor and I agreed on a course of treatment.	90.1%

#### 3.2.2. Patient satisfaction scale.

(Distribution of scores of 4 points or higher – indicating positive answers of “yes, very much” – on a 5-point scale.)

On 4 out of 6 items to measure patient satisfaction with the SDM process to determine dialysis treatment, the score distribution was over 90% with positive answers of 4 points or higher on a 5-point scale. Thus the findings indicate that patients were satisfied with the SDM process to determine dialysis method in Table 3.

**Table 3:** Patient satisfaction scale results.

Questionnaire items	4 Points or higher
1) My attending physician helped me understand all the information.	94.6%
2) My doctor knew what was important to me.	95.5%
3) My primary care physician answered all my questions.	95.5%
4) I was fully involved in the process of deciding on my treatment	89.2%
5) I decided on further treatment with my attending physician.	86.5%
6) I am satisfied with the way I discussed and made decisions about my treatment.	94.6%

#### 3.2.3. Disease awareness scale.

(Distribution of scores of 4 points or higher – indicating positive answers of “yes, very much” – on a 5-point scale.)

Among 10 items to measure disease awareness in patients in need of dialysis, the rate of responses of 4 points or higher in items 7, 8, and 10, which are positive indicators, were high at 51.3%, 46.8%, and 57.6%, respectively, while the remaining negative indicators were relatively low. Therefore, educational counseling for determination of dialysis type is shown to confirm that participating patients had high positive perceptions and low negative perceptions. Thus we conclude that SDM was effective in Table 4.

**Table 4:** Disease awareness scale results.

Questionnaire items	4 Points or higher
1) There is little I can do to improve my illness.	32.4%
2) Nothing can help my condition.	20.2%
3) My actions will have no effect on the outcome of my illness.	18.9%
4) No matter what I do, my illness will not change.	21.6%
5) My disease will only last for a short time.	11.7%
6) My illness hurts people around me.	47.7%
7) My treatment can control my illness.	51.3%
8) The negative effects of my illness may be prevented or avoided with my treatment.	46.8%
9) I do not understand my illness.	9.9%
10) There are many things I can do to control the symptoms of my illness.	57.6%

#### 3.2.4. Effects of SDM on dialysis method selection.

(Distribution of scores of 4 points or higher – indicating positive answers of “yes, very much” – on a 5-point scale.)

Out of 101 patients who received educational counseling to determine type of dialysis, 93 (92.1%) answered that SDM was helpful in selecting the best dialysis method for treatment.

#### 3.2.5. Analysis of SDM effects according to dialysis method.

##### 3.2.5.1. SDM measurement scale.

Looking at the items, “My doctor and I chose treatment together” (94% in the PD group, 87.5% in the HD group, *P* = .012) and “My doctor and I agreed on a course of treatment” (97% in the PD group, 81.3% in the HD group, *P* = .058), the group of patients that selected treatment with PD had a significantly higher percentage of positive answers of 4 points or higher in comparison to the group of patients that selected HD in Table 5.

**Table 5:** SDM measurement scale results.

Questionnaire items	Response rate of 4 points or higher (%)				*P* value
	HD	PD	KT	Undecided	
	n = 22	n = 67	n = 1	n = 11	
1) My attending physician made it clear that a decision was needed.	93.8	98.5	100	100	.240
2) My attending physician wanted to know exactly how I was involved in making the decision.	81.3	92.6	100	94.1	.752
3) My attending physician said there were several options to choose from depending on my health condition.	93.5	92.6	100	94.1	.536
4) My doctor explained in detail the pros and cons of each treatment option.	100	94.0	100	94.1	.807
5) My attending physician helped me understand the information.	100	94	100	88.2	.569
6) My doctor asked what my preferred treatment was.	93.8	91.1	100	88.3	.637
7) My doctor and I thoroughly evaluated the different options for treatment.	87.5	88.0	100	82.4	.658
8) My doctor and I chose treatment together.	87.5	94.0	100	70.6	.012
9) My doctor and I agreed on a course of treatment.	81.3	97.0	100	64.7	.058

HD = hemodialysis, KT = kidney transplantation, PD = peritoneal dialysis.

##### 3.2.5.2. Patient satisfaction.

Levels of satisfaction were higher among patients in the PD group following SDM than in patients in the HD group. Considering items including “My primary care physician helped me understand all the information” (80.6% in the PD group, 62.5% in the HD group, *P* = .005), “My primary care physician knew what was important to me” (76.1% in the PD group, 56.3% in the HD group, *P* = .019), “I was sufficiently involved in the process of deciding on my treatment” (74.6% in the PD group, 50% in the HD group, *P* = .016), and “The way I discussed and decided on my treatment” (77.6% in the PD group, 56.3% in the HD group, *P* = .010), the proportion of patients selecting PD was significantly higher than the proportion of patients selecting HD with very positive responses of 5 points to indicate patient satisfaction in Table 6.

**Table 6:** Patient satisfaction results.

Questionnaire Items	Response rate of 5 points (%)				*P* value
	HD	PD	KT	Undecided	
	n = 22	n = 67	n = 1	n = 11	
1) My attending physician helped me understand all the information.	62.5	80.6	100	47.1	.005
2) My doctor knew what was important to me.	56.3	76.1	100	35.3	.019
3) My primary care physician answered all my questions.	56.3	82.1	100	52.9	.076
4) I was fully involved in the process of deciding on my treatment.	50.0	74.6	100	41.2	.016
5) I decided on further treatment with my attending physician.	50.0	68.7	100	35.3	.075
6) I am satisfied with the way I discussed and made decisions about my treatment.	56.3	77.6	100	47.1	.010

##### 3.2.5.3. Disease awareness scale.

There were no statistical differences on the overall disease recognition scale according to dialysis method, but following SDM, PD patients tended to have higher disease awareness in Table 7.

**Table 7:** Disease awareness scale results.

Questionnaire items	Response rate of 4 points or higher (%)				*P* value
	HD	PD	KT	Undecided	
	n = 22	n = 67	n = 1	n = 11	
1) There is little I can do to improve my illness.	50.0	25.3	0	36.9	.437
2) There is nothing that can help my condition.	31.3	20.9	0	47.1	.458
3) My actions will have no effect on the outcome of my illness.	31.3	16.5	0	23.5	.076
4) No matter what I do, my illness will not change.	31.3	19.4	0	23.5	.312
5) My illness will only last for a short time.	6.3	12	0	17.6	.703
6) My illness hurts people around me.	43.8	50.8	0	41.2	.423
7) My treatment can control my illness.	50.0	50.7	0	47.1	.402
8) The negative effects of my illness may be prevented or avoided with my treatment.	50.1	49.2	0	35.3	.931
9) I do not understand my illness.	12.5	10.5	0	0	.800
10) There are many things I can do to control the symptoms of my illness.	56.3	57.3	0	47.1	.660

## 4. Discussion

### 4.1. Necessity and current status of SDM

#### 4.1.1. Necessity of SDM in patient selection of dialysis method for treatment.

Communication between clinicians and patients about treatment decisions is very important in medical situations. Much discussion about judgment and decision making in medical situations is conducted to be intentionally balanced (i.e., through SDM) for the sake of satisfying patients’ rights to knowledge and to choose. In particular, as consumer perspectives on medical services have gained traction, the importance of patient-centered communication has increased. This consumer-centered perspective affects not only the relationship between doctors and patients, but also the medical field – particularly in terms of market competition.^[1]^

Together with increases in patient participation in treatment selection and decision making, the increase in medical knowledge that patients gain through media channels in today’s media environment has had an impact on patient-centered communication. In this way, patients actively participate in treatment decisions based on information they have collected themselves. Greater involvement on the part of patients improves patient satisfaction by shared agreement on treatment methods between doctors and patients for the provision of patient-centered medical services. This encapsulates SDM.^[2]^

SDM is the basis of an existing clinical decision-making model that aims to reach final decisions through the process of comprehensive review of medical problems, patient preferences, situations, and information between medical staff and patients.^[3]^ The model for SDM can be considered a groundbreaking clinical approach wherein patients’ influence and decision-making authority are strengthened, and a departure from the conventional wisdom of patients needing to follow doctors’ instructions. SDM is characterized by the active participation of patients, with clinicians responsible for providing patients with sufficient explanations to make informed decisions related to treatment and treatment options. When this SDM is implemented, patient participation and satisfaction levels with medical processes increase. Thus the Institute of Medicine recommends the use of SDM among doctors and patients to meet a main goal of improving the quality of medical services.^[4,5]^

As societies continue to age, chronic diseases such as high blood pressure and diabetes are increasing. As a result, CKD is increasing together with the socioeconomic burden of managing long-term disease. CKD is the second most expensive disease after high blood pressure, and accounts for about 3% of total medical expenses in certain populations in Korea. When progressing to end-stage renal failure, medical costs for HD patients increased from 299 billion won in 2003 to 1.44 trillion won in 2015.^[6]^ Likewise, medical costs for PD patients increased from 76 billion won to 160 billion won during the same period. As CKD worsens and progresses to end-stage renal disease, patients must consult with medical staff to determine appropriate renal replacement therapies. In 2019, the number of dialysis patients in Korea was about 84,045, which is a 39-fold increase since the first count in 1986.^[7]^

For patients with CKD who require dialysis, both medical staff and patients need to consider the patient’s right to choose a dialysis method that is considered appropriate for the patient by sharing sufficient information. In particular, due to the specificity of dialysis treatment (HD requires treatment at a medical institution 3 times a week for 4 hours each time, while PD requires hand dialysis or machine dialysis at home every day), patient understanding and SDM between doctors and patients are essential to determine the right treatment fit. Based on the characteristics of diseases that require long-term, continuous relationships with medical staff, it is necessary to establish SDM to improve quality of life for patients with chronic diseases, including chronic kidney disease.

SDM is a two-way communication that includes both doctors and patients, and is balanced in its collection of opinions based on mutual respect and authority. Two-way communication between doctors and patients has 2 purposes – namely, information exchange and relationship formation – and as a result, treatment effects are enhanced and high levels of patient satisfaction are experienced.^[8]^ Other positive effects of SDM are to reduce various side effects of medical negligence by providing information to medical staff to actively guarantee and defend the rights of patients and to make efforts to prevent possible harm to them.^[9]^ In addition, SDM has been found to be more effective than traditional methods for patients with chronic diseases confronting long-term decisions or requiring multiple treatments.^[10]^

Recent clinical studies on SDM verify these results. Research in SDM conducted through telephone interviews with patients with advanced CKD demonstrates no difference in the HD/PD selection ratio between patients.^[11]^ A study to investigate oral or video education of elderly patients aged 65 years or older with advanced CKD does not show any improvement in their knowledge of dialysis options, but the video-educated patients are shown to have high levels of satisfaction.^[12]^ In a study on patients’ experience and decision quality following SDM to determine dialysis type, more than 80% of participating patients reported that they thoroughly understood the dialysis treatment methods and felt they were ready to make informed decisions for dialysis treatment.^[13]^ Other research in SDM for the treatment of advanced CKD suggests that a more holistic and patient-centered approach is necessary for success.^[14]^ A comparative study in SDM for treatment selection concludes that evaluation of SDM is high in PD patients and that information on renal replacement therapy should be provided to patients in the early stages of CKD.^[15]^

A Korean study on the development and effectiveness of an SDM-based self-reported questionnaire for determining type of dialysis in patients with CKD shows that the approach allows CKD patients to choose treatments that closely match their own values. Concluding that it could help both clinicians and patients to make informed, shared decisions, this research was the first to develop and validate items to help determine dialysis types for patients with CKD.^[16]^

#### 4.1.2. SDM.

SDM for renal replacement therapy refers to decision making through an in-depth discussion process between patients and medical staff based on patients’ values and preferences. SDM can help patients lead healthy and happy lives through planned dialysis by guaranteeing patients’ right to know and choose, and by providing them with sufficient education to improve the quality of medical care. The process of SDM is as follows.

**Figure 1.**
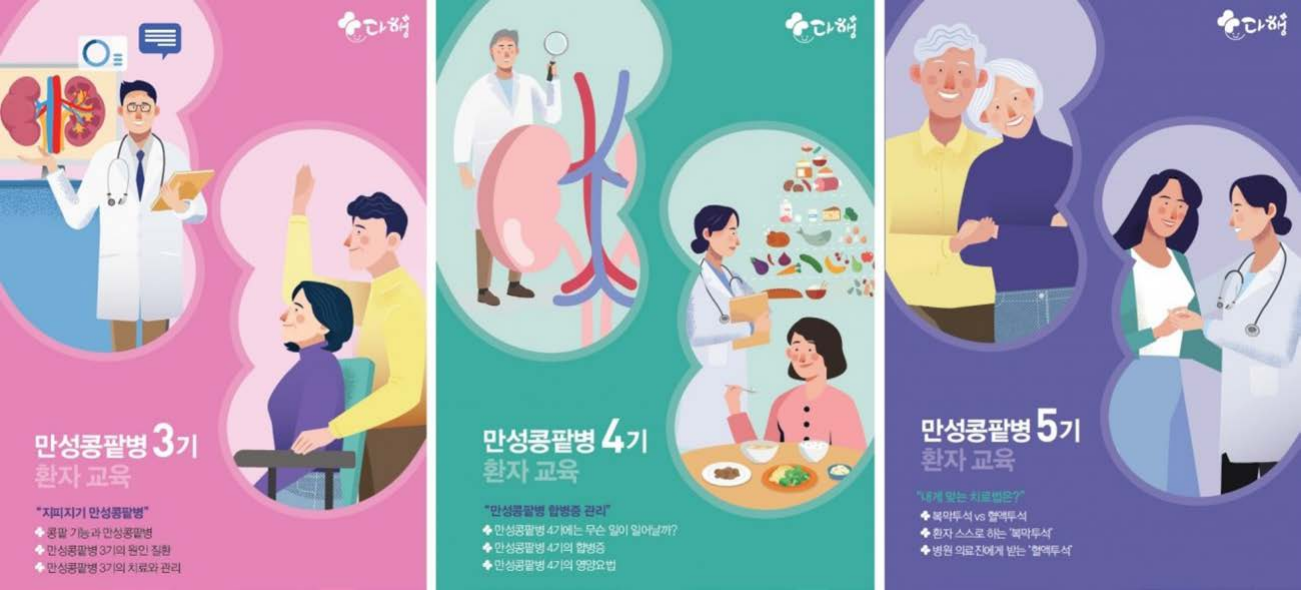
Data for patient distribution according to stage of chronic kidney disease.

**Figure 2.**
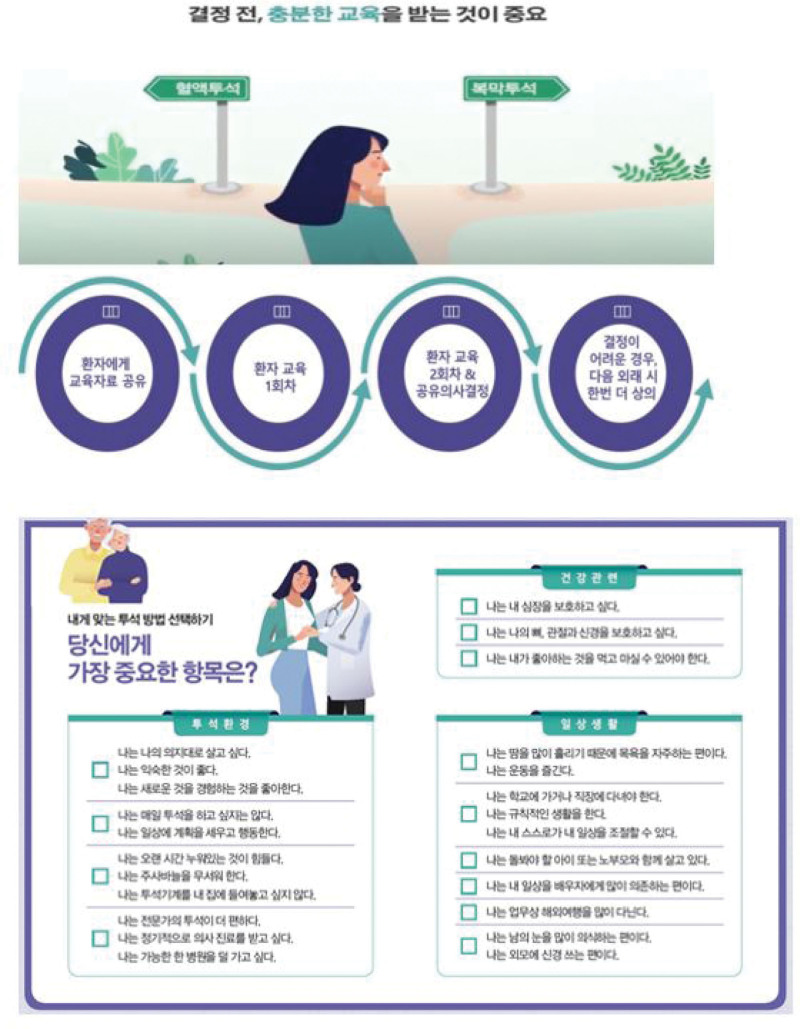
Educational videos to facilitate patient understanding.

First, educational materials for patients in need of SDM for dialysis decisions (e.g., educational booklets comparing various dialysis methods and video materials introducing SDM, HD, PD, and other topics – see Figs.1 and 2) are provided for initial training.

Second, medical staff and patients share opinions about types of dialysis based on this initial education.

① A pre-dialysis patient with renal failure who has decided to start dialysis is invited to participate in a shared decision-making dialogue. Wherever possible, a guardian should accompany the patient to participate in decision making.
② Medical staff inform patients of available dialysis types (HD or PD).
③ Before dialysis, the patient discusses their initial thoughts on different dialysis methods in the process of SDM.

Third, doctors and patients share opinions on options for dialysis methods.

① Discussion is conducted after a patient has thoroughly reviewed the data on a dialysis decision provided to him/her.
② Medical staff conducts interviews in consideration of the patient’s most important value (e.g., lifestyle choice) related to treatment.
③ Medical staff and patients talk about any information that is difficult for patients to understand.
④ Following consultation, a patient is introduced to the specific process after deciding on a method of dialysis.

**Figure 3.**
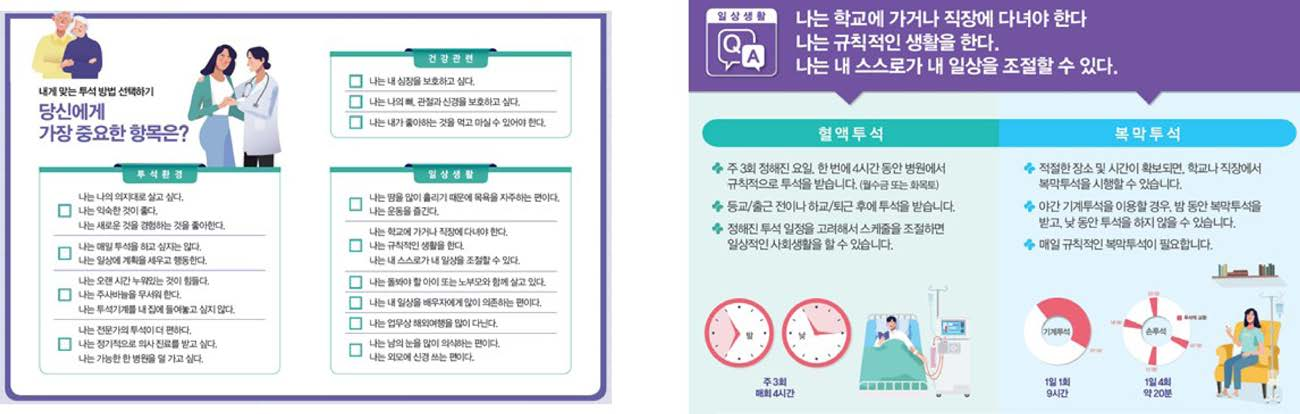
Self-diagnosis tool for patient consultation and explanation in calendar format.

To determine the type of dialysis, medical staff and patients share their opinions (Fig. 3).

① Medical staff and pre-dialysis patients review the interviews conducted so far to check whether patients’ situations or values have changed.
② Both medical staff and patients review the process so far to identify any potential problems.
③ Patients are prepared to make an informed final decision about the dialysis regimen.
④ Medical staff provide full support for patients’ individual decisions and initiate measures so that treatment can begin.
  ○ During the SDM process, in order to select the most suitable dialysis method, patients respond to the questionnaire shown in Table 8.

**Table 8:** Choosing the right dialysis method for you.

What is most important to you?
1. Health-related considerations I want to protect my heart. I want to protect my bones, joints, and nerves. I want to live according to my will. I do not want to be a burden to my family. I want to keep my quality of life as high as possible. I want to spend a day without the burden of dialysis. I do not want to go on dialysis every day. I want to see a doctor regularly. I can control my daily routine by myself. I lead a regular life. I plan and act in my daily life. I enjoy exercising. 2. Dialysis environment I want to go to the hospital as little as possible. I am more comfortable with expert dialysis. I like being in a familiar environment. I hate environmental changes. I like to experience new things. I like watching TV. I find it difficult to lie down for long periods of time. I am afraid of needles. I tend to be very conscious of other people’s eyes. I tend to depend a lot on my spouse for my daily life. I need someone to take care of me. I do not want a dialysis machine in my house. 3. Daily life I want to spend as much time as possible with my family. I live with and take care of my elderly parents. I am raising a child. I have to go to school or go to work. I should be able to eat and drink what I like. I sweat a lot, so I shower or bathe often. I tend to care about my appearance. I take a lot of time to get used to new things. I have no one to take care of me. I love to travel and never give up. I travel abroad a lot.

## 5. Conclusion

Among participating patients, SDM was shown to be effective in making decisions on dialysis treatment, and patients were shown to be satisfied with the dialysis method decision process. On the disease awareness scale, those who participated in this project had relatively high positive and low negative perceptions, further verifying that SDM was relatively effective. Implementation of SDM was helpful for patients selecting a dialysis method, while SDM scale results were higher among patients in the PD group than in the HD group.

Analysis of the effects of SDM on clinical indicators and patient prognosis shows decreases in periods of hospitalization, in hospitalization costs, and in the use of phosphorus binders by patients.

Through SDM, patients are able to select an appropriate method of dialysis and feel satisfied with the dialysis treatment decision-making process. SDM is shown to have positive effects on clinical indicators (reduction of hospitalization periods, reduction of hospitalization costs, and reduction of the use of phosphorus binders by patients).

## Author contributions

**Conceptualization:** Young-Ki Lee, Ho Sik Shin.

**Data curation:** Jin-Heog Kim, Won-Min Hwang.

**Formal analysis:** Jin-Heog Kim.

**Investigation:** Do-Hyoung Kim, Jeong-Hwan Lee, Ji Hyeon Park, Gang-Jee Ko, Won-Min Hwang, Hyo-Wook Gil, Young-Sun Kang, Kyu-Bok Jin, Jun-Young Do, Se-Joong Kim, Beom-Seok Kim.

**Methodology:** Hyo-Wook Gil.

**Resources:** Ji Hyeon Park.

**Writing – original draft:** Yang-Hyeon Kim, Gang-Jee Ko, Ho Sik Shin.

**Writing – review & editing:** Yang-Hyeon Kim, Young-Sun Kang, Ho Sik Shin.
